# The value of informal care in the context of option B+ in Malawi: a contingent valuation approach

**DOI:** 10.1186/s12913-016-1381-y

**Published:** 2016-04-19

**Authors:** Levison Stanely Chiwaula, Gowokani Chijere Chirwa, Fabian Caltado, Atupele Kapito-Tembo, Mina C. Hosseinipour, Monique van Lettow, Hannock Tweya, Virginia Kayoyo, Blessings Khangamwa-Kaunda, Florence Kasende, Clement Trapence, Salem Gugsa, Nora E. Rosenberg, Michael Eliya, Sam Phiri, For PURE Malawi Consortium

**Affiliations:** Department of Economics, University of Malawi, P.O. Box 280, Zomba, Malawi; Dignitas International, Zomba, Malawi; College of Medicine, University of Malawi, Blantyre, Malawi; University of North Carolina Project, Lilongwe, Malawi; Lighthouse Trust, Lilongwe, Malawi; Department of HIV/AIDS, Ministry of Health, Lilongwe, Malawi

**Keywords:** Informal care, Willingness to Pay, Willingness to Accept, PMTCT, Option B+, Malawi

## Abstract

**Background:**

Informal care, the health care provided by the patient’s social network is important in low income settings although its monetary value is rarely estimated. The lack of estimates of the value of informal care has led to its omission in economic evaluations but this can result in incorrect decisions about cost effectiveness of an intervention. We explore the use of contingent valuation methods of willingness to pay (WTP) and willingness to accept (WTA) to estimate the value of informal care provided to HIV infected women that are accessing antiretroviral therapy (ART) under the Option B+ approach to prevention of mother-to-child transmission (PMTCT) of HIV in Malawi.

**Methods:**

We collected cross sectional data from 93 caregivers of women that received ART care from six health facilities in Malawi. Caregivers of women that reported for ART care on the survey day and consented to participate in the survey were included until the targeted sample size for the facility was reached. We estimated the value of informal care by using the willingness to accept (WTA) and willingness to pay (WTP) approaches. Medians were used to summarize the values and these were compared by the Wilcoxon signed-rank test.

**Results:**

The median WTA to provide informal care in a month was US$30 and the median WTP for informal care was US$13 and the two were statistically different (*p* < 0.000). Median WTP was higher in the urban areas than in the rural areas (US$21 vs. US$13, *p* < 0.001) and for caregivers from households from higher wealth quintile than in the lower quintile (US$15 vs. US$13, *p* < 0.0462).

**Conclusion:**

Informal caregivers place substantial value on informal care giving. In low income settings where most caregivers are not formally employed, WTP and WTA approaches can be used to value informal care.

**Clinical trial number:**

NCT02005835.

**Electronic supplementary material:**

The online version of this article (doi:10.1186/s12913-016-1381-y) contains supplementary material, which is available to authorized users.

## Background

Informal care is the health care that is provided by family, friends, acquaintances, or neighbours of a patient for which they do not have to be financially compensated [1, 2]. The time contributed by informal caregivers to provide care reflects a cost in the form of forgone income or forgone leisure because care is mostly provided out of obligation [2]. Unfortunately, information on the cost of providing informal care to people living with HIV (PLHIV) in Africa is not readily available and there are many calls for the generation of this information for probable integration of it in economic evaluations of healthcare interventions adopting the societal perspective [3–5]. Available informal care costing studies are either conducted for diseases different from AIDS or in regions outside Africa [6–8]. Lack of such studies in Africa may be due to methodological challenges. Most methodological papers were developed in high income countries [5, 7–11] using methods that cannot be directly utilized in low income settings. Revealed preference methods of opportunity cost and proxy good methods are the commonly used approaches to value informal care. The opportunity cost method values informal care time by using the market wage of the caregiver while the proxy good method values informal care time by using the market price of a close substitute [3]. Application of the revealed preference methods are challenging in low income countries because of the problems with measurement of time and its valuation. Measurement of time is problematic because the concept of time in low income countries can be slightly different to that in Europe or America. Concept of time in subsistence societies such as in Africa is attached to changes in nature such as cock crow, sun rise, midday meal, etc. and not necessarily hours and minutes [12, 13]. Other problems of the commonly used methods of measuring time are highlighted elsewhere [3, 7]. Valuation of informal care time by using wage rates is challenging in low income countries because a substantial proportion of caregivers are subsistence producers and therefore don’t receive a market wage [6]. Additionally, opportunity cost method and the proxy good method may neglect the full impact of providing informal care on the caregiver. Providing informal care involves burden, morbidity risks and in some cases even mortality risks [3, 14, 15].

We therefore attempt to estimate the cost of providing informal care to women that have been enrolled on antiretroviral therapy (ART) within Option B+ approach of the prevention of mother-to-child transmission (PMTCT) in Malawi. Option B+ is a public health approach to promote maternal health and eliminate paediatric HIV infections through a “test and treat” strategy [16, 17]. The strategy offers all HIV-infected pregnant and breastfeeding women lifelong ART regardless of CD4 count or World Health Organisation (WHO) clinical stage [16, 17]. This enables more women that have not developed AIDS symptoms to start receiving antiretrovirals (ARVs). Malawi adopted the ‘Option B+’ in 2011 following the 2010 WHO guidelines. Valuing informal care is important in this population because it involves people who would primarily be caregivers themselves and it would good it assess if they receive care when they are in need. We used the willingness to pay approach to estimate the cost as a way of dealing with the problem of unobserved market wages.

## Methods

In this study, we used a modified diary that divided a day into a number of natural changes such as waking up, sunrise, main meals, sunset and sleeping. Respondents were required to indicate the activities they carried out according to the approximate time of day [6, 18].

### Contingent valuation approach to informal care valuation

The paper used stated preference methods of contingent valuation method (CVM) to elicit values of informal care to overcome the challenges of using revealed preference methods in valuation of informal care in low income countries. CVM is a survey-based, hypothetical and indirect method of determining the monetary valuations associated with the provision of goods and services [19] by eliciting willingness to pay (WTP) or willingness to accept (WTA) a good or service by its users. The methods involve asking the caregivers about the minimum amount of money they would be willing to receive if they are to provide an additional amount of care [3] or the maximum amount of money they are willing to pay for somebody to provide care. In this study, WTA was elicited by asking the caregivers the question:

*Suppose there is a possibility for you to provide care to somebody you are not related to for one month and the government is willing to pay you for the care you will provide. What is the minimum amount of money you would be willing to accept to provide the care?*

The WTP was elicited by asking the caregivers the question:

*Suppose you become too busy to provide care to your recipient and you have found somebody who is willing to be paid for him/her to provide care to your client, what is the maximum amount of money you would be willing to pay the individual per month?*

WTA questions were directed to a stranger because caregivers are mostly unwillingness to associate caregiving with problems as this will culturally be considered as a sign of insufficient love or not wishing the patient [14]. When asked to attach a value to additional care they provide to their loved ones, caregivers may give protest responses.

### Setting and study sites

This study was implemented as part of the formative research of PMTCT Uptake and REtention (PURE) Clinical trial in Malawi. The PURE trial was one of the six trials supported by the Department of Foreign Affairs, Trade and Development Canada (DFADT) through World Health Organisation—Integrating and Scaling up PMTCT though Implementation Research (INSPIRE) Projects [20]. The trial was designed to evaluate facility-based and community-based support models of providing PMTCT care to strengthen uptake and retention of mothers and families in PMTCT care in Malawi. The overall trial hypothesis is that enhanced support for women and their families within facilities and/or through community outreach will result in improved retention in the continuum of PMTCT care [21]. The aims of the formative research for the PURE trial were to collect baseline information about the provision of PMTCT services; help to define the nature and scope of each intervention arm; and serve as a baseline for the qualitative and economics components of the trial.

The study was conducted in three health zones of Malawi, namely South East, South West and Central West zones. The six health facilities were purposively selected from a list of 21 facilities that qualified to be in PURE trial [21]. The six facilities were selected based on their variability in geographical location, level of service provision, ownership, and size of the facility.

### Study design and data collection

We purposively decided to collect data from 15 care recipients and their care givers per health facility. The study involved six health facilities that were purposively selected from a list of 21 facilities that qualified for inclusion in the trial [21]. At each health facility, health care workers identified women that were enrolled in Option B+. All identified women were requested to participate in the study until the sample size was reached. Women who consented to participate in the study were interviewed and asked to identify their primary caregivers that were also interviewed. We targeted primary caregivers only because we assumed that these are the individuals that provided most of the informal care. Data was collected using a semi structured questionnaire between March and April 2013 through face to face interviews. The final sample size was 93 (Table 1 and Additional file 1).

**Table 1:** Summary health facilities and sample sizes

Name of Facility	Health Zone (Location)	Level of Service	Ownership	Location	Sample Size
Mulanje DH	South East	Secondary	Public	Rural	15
Muloza HC	South East	Primary	Public	Rural	16
Nsipe HC	Central West	Primary	CHAM	Rural	15
Lobi RH	Central West	Primary	Public	Rural	16
Trinity MH	South West	Secondary	CHAM	Rural	15
Makhetha HC	South West	Primary	Public	Urban	16

Note: *DH* District Hospital, *HC* Health Centre, *RH* Rural Hospital, *MH* Mission Hospital

### Statistical analysis

Data analysis involved determination of household socioeconomic status and assessment of the health related quality of life (HRQoL) of PLHIV and their caregivers, and the assessment of the relationship between socioeconomic status and health outcomes with the value of informal care. Household socioeconomic status was derived by using an asset index that was derived by using the multiple correspondence analysis [22]. The households were categorized into two groups depending on their position on the wealth status. EQ5D was used to measure the HRQoL. The EQ5D assesses health status on five domains of mobility, self-care, usual activities, pain/discomfort and anxiety/depression at three levels which include no problem, some problems, and extreme problems/unable to perform task [23]. Caregivers and PLHIV were considered to have a perfect health state if they reported to have no problems with all the five domains of the EQ5D and imperfect if they reported a problem in at least one of the domains. We used descriptive statistics of medians and interquartile range (IQR) to analyse the data because our sample is small. The differences in the medians were tested by using the Wilcoxon signed-rank test. Data analysis was conducted in Stata version 13.

## Results

### Characteristics of informal caregivers

The descriptive characteristics of the informal caregivers and informal care recipients w presented in Table 2. Participation rate was 93/93(100 %) for both patients and guardians. The findings show that most of the informal caregivers were male and the majority of them were husbands of care recipients. Care giving was also provided by mothers, siblings, in laws and other family members or neighbours. The median age of informal caregivers was 38 years and that of care recipients was 28 year. Most of caregivers 69/93(74 %) and care recipients 61/93(66 %) were married. The findings also show that majority of caregivers 71/93 (79 %) and care recipients 76/93(82 %) had no or primary level education. Most of the caregivers and care recipients obtained their incomes from farming and petty trading. The median monthly income for caregivers was MK2720 (~US$6.80).

**Table 2:** Characteristics of informal caregivers and care recipients

Variable		Informal caregiver		Informal care recipients	
	Characteristic	n	Statistic	n	Statistic
Sex (%)	Male	66	71	0	0
	Female	27	29	93	100
Relationship with care recipient					
	Husband (%)	49	53		
	Mother	14	15		
	Daughter	3	3		
	Sibling	9	10		
	In law	10	11		
	Other	8	9		
Age	Median (IQR)	93	38 (32,51)	93	28(23, 31)
Marital Status (%)					
	Married	69	74	61	66
	Widowed	14	15	9	10
	Separated	10	11	20	21
	Never Married	0	0	3	3
Level of Education (%)					
	None	14	15	7	8
	Primary	59	64	69	74
	Secondary	19	20	16	17
	Post-secondary	1	1	1	1
Major Source of Income (%)					
	Permanent employee	8	9	0	0
	Temporary employee	11	12	1	1
	Farmer	32	34	26	28
	Small businesses	30	32	38	41
	Casual work	8	9	16	17
	Other	3	3	12	13
Monthly income (Median)		93	-	93	2720 (1000, 5000)

### Amount of informal care time

Although all the 93 informal caregivers were identified by the women receiving care, only 56 % (52/93) of them were involved in activities that were defined as informal care in this study: collecting drugs for the patient, escorting the patient to the health facility, providing encouragement and counselling to the patient. Other activities such as washing clothes, preparing meals, and collecting water were not defined as informal care in this study because some of these are done to other members of the family even in the absence of HIV infection. The median values of informal care are thus provided for caregivers that reported to have supplied positive informal care hours. Data on informal caregiving activities and time is presented in Table 3.

**Table 3:** Median of informal care time provided to women on Option B+ in month

Informal care activity	Frequency	Percent	Median (IQR)	Mean (Std Dev)
Collecting drugs for the women	1	1.1	0.0 (0.0, 0.0)	0.0 (0.0)
Escorting the women to health facility	3	3.2	0.0 (0.0, 0.0)	0.7(5.1)
Providing encouragement to the women	52	55.9	0.8 (0.0, 16.0)	17.2(34.2)
Total informal care time	52	55.9	0.8 (0.0, 16.0)	17.9 (37.5)

The median informal care time was about 0.8 h in a month while the mean was 18 h. Most of informal care provided to women on Option B+ was in the form of encouragement to the mothers.

### WTP and WTA

The estimated median WTA to provide informal care was US$30.0 (US$13.8, US$50.0) while the median WTA for informal care was US$12.5 (US$7.5, US$25.0)) and the two were statistically different (*p* < 0.000). These were also estimated for different sub-groups of informal carers and the results are presented in Table 4.

**Table 4:** Median values of willingness to accept and willingness to pay for informal care (US$ per month)

Characteristics	Frequency	Willingness to Accept	Willingness to Pay
Male	66	38	13
Female	27	30	13
p-value		0.218	0.1682
Rural	77	25	13
Urban	16	50	21
p-value		0.0320	0.0001
Care Recipient in Perfect HRQoL	48	30	13
Care recipient in Imperfect HRQoL	45	34	13
p-value		0.5613	0.8713
Carer in Perfect HRQoL state	56	28	13
Carer in Imperfect HRQoL state	27	38	13
p-value		0.9551	0.8430
Lower socioeconomic status	47	25	13
Higher socioeconomic status	46	38	15
p-value		0.0932	0.0462
All Observations	93	30 (17.5, 56.3)	12.5(7.5,25)**

Note: ** p-value for difference between WTA and WTP = 0.000Exchange rate at time of survey: 1 USD = MK400

The median WTA was larger than the median WTP for the different caregiver sub-groups. The median WTP for most of the caregiver sub-groups was around US$13 while the median WTA for different caregiver sub-groups varied between US$25 and US$50. The median WTA and WTP for informal care for caregivers from urban areas and caregivers in the higher wealth quintile are were larger than the for caregivers from the rural areas and in the lower wealth quintiles, respectively. There was no difference in the WTA and WTP for male and female caregivers.

## Discussion

We estimated informal care time and value provided to women receiving ARVs under Option B+ in Malawi. In our sample most of the caregivers are husbands of the care recipients. This makes our sample different from other studies that have shown that women are principal providers of informal care [2, 18] because they are culturally expected to do so. This finding suggests that when women, who are culturally principal informal caregivers are in need of care, husbands are the likely providers of informal care

We also found that 66 % were self-employed through faming or businesses. This creates flexibility on the caregivers to allocate time to informal care giving. However, the high proportion of self-employed caregivers also creates problems in determining the wage rates when we are using the opportunity cost method of valuing informal care, thereby supporting the approaches we used.

The median value of informal care provided to women in PMTCT under the Option B+ is US$13 per month when we use the willingness to pay approach and US$30 per month when we use the willingness to accept approach. The observed difference in the median values of informal care estimated by the WTA and WTP is not unique to our study. Studies that have estimated both WTA and WTP for health services in general [22, 23] and for informal care [11] have also established this pattern. We however note that the magnitude in the differences may also be influenced by the use of different reference care recipients in the questions—loved one for WTP and stranger for WTA. In responding to willingness to pay questions, caregivers consider their income generation opportunities. The informal care value that has been derived through the WTA approach may be biased upwards because they reflect a potential income to the caregivers. We therefore considered the value derived from WTP to be more realistic. Other studies have also placed more weight on WTP [23].

The estimated values of informal care are comparable with values derived in other studies although the settings and diseases are different. For example, Ama and Seloilwe [18] in Botswana estimated the value of informal care time for people living with HIV and AIDS at US$25 per month while Van den Berg et al. [11] used the WTP and WTA approaches to estimate the value of informal care to be between €7(~US$6.27) and €11 (~US$9.86) per month. Our view is that provision of informal care is obligatory in African context such that caregivers would provide care because society is expecting them to do so [2]. The obligatory nature of care giving implies that caregivers would have been working in productive activities or enjoying leisure had they not provided informal care. The estimated values of informal care are also substantially higher than the monthly median incomes of care recipients which was estimated at US$6.80. The estimates we found are therefore suggesting that caregivers forgo a substantial amount of income in form of productive time and leisure. These estimates suggest that inclusion and exclusion of informal care in cost effectiveness analyses of PMTCT programmes in general and Option B+ in particular would result in different conclusions.

Caregivers from urban centres and those from higher wealth quintile had higher values of informal care than those from rural areas and lower wealth quintile. Using regression analysis, van den Berg et al. [11] also found high values of informal care for high income households. High values of informal care for urban dwellers and households in high socioeconomic groups reflect the high ability to pay for those households. This is consistent with economic theory because we expect the individuals from high income families to have high opportunity cost of time. The health related quality of life of the care recipient and the caregiver has not been observed to significantly relate to the elicited values of informal care.

The results in this study seem to be within the ranges of other informal care valuation studies. However, we are not confident to strictly compare our findings with these estimates because the settings and contexts are not similar. This is due to the fact that there aren’t many studies that have valued informal care for similar populations and in similar settings.

## Conclusions

Informal caregivers of women that are receiving ARVs under the Option B+ bears cost in the form of forgone productive time and leisure. In low income settings where formal employment is rare, these can be estimated by using willingness to pay or willingness to accept approaches. The estimated values should be considered in cost effectiveness analysis of PMTCT programmes because their exclusion may result in misleading conclusions..

### Ethics

Ethical approval for the research was obtained from the Malawi’s National Health Sciences Research Committee (NHSRC), the University of North Carolina Institutional Review Board, WHO Ethics Review Committee and the University of Toronto Institutional Review Board.

### Consent to participate

Survey participants provided written informed consent.

### Availability of data and materials

Data that are supporting our findings have been made available as an Additional file.

## Additional file

Additional file 1: Data used in the analysis. (XLSX 130 kb)

## Abbreviations

ART: antiretroviral therapy
ARVs: antiretrovirals
CVM: contingent valuation method
DFADT: Department of Foreign Affairs, Trade and Development Canada
HRQoL: health related quality of life
INSPIRE: INtegrating and Scaling up PMTCT though Implementation Research
IQR: interquartile range
NHSRC: National Health Sciences Research Committee
PLHIV: people living with HIV
PMTCT: prevention of mother-to-child transmission
PURE: PMTCT Uptake and REtention
WHO: World Health Organisation
WTA: willingness to accept
WTP: willingness to pay
