# Erratum to: The value of informal care in the context of option B+ in Malawi: a contingent valuation approach

**DOI:** 10.1186/s12913-017-2130-6

**Published:** 2017-03-10

**Authors:** Levison Stanely Chiwaula, Gowokani Chijere Chirwa, Fabian Cataldo, Atupele Kapito-Tembo, Mina C. Hosseinipour, Monique van Lettow, Hannock Tweya, Virginia Kayoyo, Blessings Khangamwa-Kaunda, Florence Kasende, Clement Trapence, Salem Gugsa, Nora E. Rosenberg, Michael Eliya, Sam Phiri, For PURE Malawi Consortium

**Affiliations:** 1 Department of Economics, University of Malawi, P.O. Box 280, Zomba, Malawi; 2 Dignitas International, Zomba, Malawi; 3 College of Medicine, University of Malawi, Blantyre, Malawi; 4 University of North Carolina Project, Lilongwe, Malawi; 5 Lighthouse Trust, Lilongwe, Malawi; 6 Department of HIV/AIDS, Ministry of Health, Lilongwe, Malawi

## Erratum

After publication of the original article [1] it was brought to our attention that author Fabian Cataldo was incorrectly included as Fabian Caltado. The correct spelling of the name is included in the author list of this erratum.
